# Ventricular assist device implantation in patients with a failing systemic right ventricle: a call to expand current practice

**DOI:** 10.1007/s12471-019-01314-y

**Published:** 2019-08-16

**Authors:** T. E. Zandstra, M. Palmen, M. G. Hazekamp, B. Meyns, S. L. M. A. Beeres, E. R. Holman, P. Kiès, M. R. M. Jongbloed, H. W. Vliegen, A. D. Egorova, M. J. Schalij, L. F. Tops

**Affiliations:** 1 Department of Cardiology, Leiden University Medical Center, Leiden, The Netherlands; 2 Department of Cardiothoracic surgery, Leiden University Medical Center, Leiden, The Netherlands; 3 Department of Cardiothoracic surgery, University Hospital (UZ) Leuven, Leuven, Belgium; 4 Department of Anatomy & Embryology, Leiden University Medical Center, Leiden, The Netherlands

**Keywords:** Cardiac assist devices, Heart failure, Heart defects, Congenital

## Abstract

Ventricular assist device (VAD) implantation is an established treatment modality for patients with end-stage heart failure, and improves symptoms and survival. In the Netherlands, it is not yet routinely considered in patients with congenital heart disease and failing systemic right ventricle (SRV). Recently, a VAD was implanted in 2 SRV patients, one who underwent a Mustard procedure during infancy for transposition of the great arteries (male, 47 years old) and one with a congenitally corrected transposition of the great arteries (male, 54 years old). The first patient is doing well >1 year after implantation; the second patient will be discharged home soon. These examples and other reports demonstrate the feasibility of adopting VAD implantation into routine care for SRV failure. In conclusion, patients with SRV failure may be suitable candidates for VAD implantation: they are relatively young, usually have a preserved subpulmonary left ventricular function, and their specific anatomical and physiological characteristics often make them unsuitable for cardiac transplantation. Therefore it is important to recognise the possibility of VAD implantation early in the process of SRV failure, and to timely refer these patients to a heart failure clinic with experience in VAD implantation in this group of patients for optimisation, screening, and implantation.

## Current use of ventricular assist device therapy and gap for patients with failing systemic right ventricle

Left ventricular assist device (LVAD) implantation as destination therapy is an established treatment for patients with end-stage heart failure who are not eligible for cardiac transplantation. It improves both symptoms and prognosis [1]. However, in the Netherlands, it has until recently not been used as a treatment option for congenital heart disease (CHD) patients with failing systemic right ventricle (SRV). This group includes patients late after Mustard or Senning procedure for transposition of the great arteries (TGA) or patients with congenitally corrected TGA (CCTGA). Current survival of Mustard/Senning patients is 82% at 40 years postoperatively [2]. For CCTGA patients, freedom from death or cardiac transplantation was 84% at 40 years of follow-up [3]. SRV failure is likely to be a major and substantial problem in the upcoming years [2, 3]; in our centre alone, 61 SRV patients are currently under follow-up. SRV patients have a complex anatomy, adhesions due to (sometimes multiple) prior sternotomies, and pulmonary hypertension, and are consequently likely to be rejected for cardiac transplantation due to current shortage of donor organs. The European Society of Cardiology (ESC) guideline for adult CHD does not yet contain an advice regarding VAD implantation but mentions long-term mechanical circulatory support as an important area of research [4]. Recently, we implanted a VAD in 2 SRV patients. In this paper we aim to illustrate the feasibility of this procedure, to stress the clinical necessity to expand current indications for VAD therapy to this group, and especially to consider it as destination therapy.

## Cases of VAD implantation in SRV: clinical and surgical considerations

**Fig. 1:**
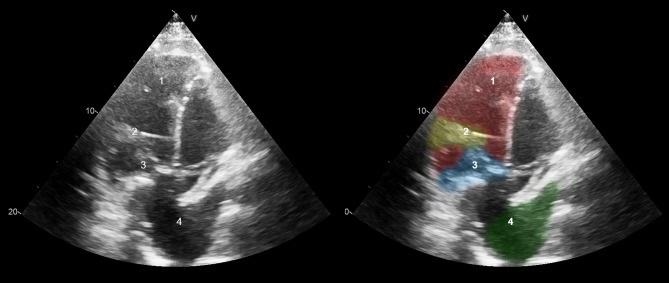
Transthoracic echocardiography of patient 1 after VAD implantation 1 systemic right ventricle 2 inflow cannula 3 tricuspid valve prosthesis 4 pulmonary venous tunnel

**Fig. 2:**
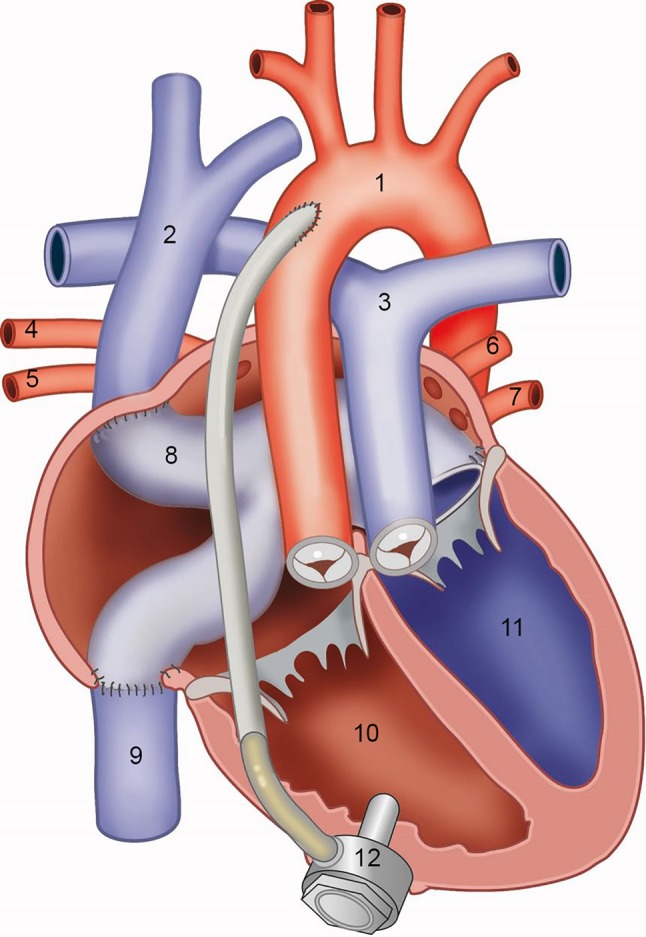
Anatomy of patient 1 after VAD implantation 1 aorta 2 vena cava superior 3 pulmonary trunk 4 right superior pulmonary vein 5 right inferior pulmonary vein 6 left superior pulmonary vein 7 left inferior pulmonary vein 8 baffle 9 vena cava inferior 10 systemic right ventricle 11 subpulmonary left ventricle 12 ventricular assist device

The first patient is a 47-year-old man late after Mustard procedure for TGA. The tricuspid valve (systemic atrioventricular valve) was replaced two years before VAD implantation because of severe regurgitation. After tricuspid valve surgery he developed symptoms of advanced heart failure (New York Heart Association [NYHA] class IIIb) despite optimal medical therapy. He was screened for cardiac transplantation and rejected due to pulmonary hypertension (mean pulmonary artery pressure 29.7 mm Hg, transpulmonary gradient 12.7 mm Hg, estimated pulmonary vascular resistance 4.2 Woods Units). SRV function was poor (2D global longitudinal strain [GLS] −4.7%, fractional area change [FAC] 9.2%). The subpulmonary left ventricular function was reasonable. The patient showed advanced symptoms of heart failure and, consequently, was screened and accepted for VAD implantation. Pre-operatively, the patient was optimised with inotropic support and was in INTERMACS (Interagency Registry for Mechanically Assisted Circulatory Support) level 3 at the time of surgery. Through median re-sternotomy and with cardiopulmonary bypass, a VAD (HVAD, Medtronic, USA) was implanted in the SRV after resection of multiple trabeculations in the SRV cavum. Because of anatomical considerations, the VAD was positioned mid-basally instead of apically, which is common for VAD implantation in the left ventricle (Figs. 1 and 2). Postoperative transoesophageal echocardiography (TEE) demonstrated normal VAD inflow and outflow signals and good VAD performance. Recovery was uneventful for 13 days. Then, a re-operation was necessary because of cardiac tamponade; following re-operation patient recovered well. Shortly after discharge, the patient suffered a haemodynamically tolerated sustained monomorphic ventricular tachycardia (185/min), probably originating from the surgical scar, which was terminated with procainamide. Eight months postoperatively, an ischaemic stroke occurred under clopidogrel and an adequate international normalized ratio (INR), with mild cognitive sequelae. A risk factor in this may have been the aortic valve, which showed reduced opening after VAD implantation. His target INR was raised. His maximum workload (measured with bicycle ergometry) is still improving from 70 Watts pre-implantation, to 80 Watts after 6 months of VAD support, and to 90 Watts currently. More than 1 year postoperatively, the patient is doing well and functioning in NYHA class II.

The second patient is a 54-year-old man with CCTGA, who underwent tricuspid valve replacement with a bioprosthesis and mitral valve annuloplasty 2 years before VAD implantation, the latter of which was complicated by partial ring dehiscence. The defect was closed percutaneously with 2 vascular plugs. In 2017, he received an ICD for primary prevention because of a poor SRV function. Recently, his clinical condition deteriorated rapidly and he was admitted because of congestion. He was rejected for cardiac transplantation because of renal dysfunction and, consequently, screened and accepted for VAD implantation. Transthoracic echocardiography (TTE) confirmed the poor SRV function (GLS −2.0%, FAC 7.3%). Pre-operative admission was prolonged due to biliary pancreatitis, which was treated with laparoscopic cholecystectomy. At the time of surgery, the patient was in NYHA class IV, INTERMACS level 2. Because of a decline in subpulmonary left ventricular function, treatment with levosimendan (Orion Corporation) was initiated. The VAD was implanted through a left-sided anterolateral thoracotomy combined with upper hemisternotomy because of a relatively dorsolateral position of the SRV and favourable anatomy for this approach. Again, multiple trabeculations were resected in the SRV cavum before the VAD (HVAD, Medtronic, USA) was implanted. Early after surgery, VAD flow dropped due to a deviation of the inflow cannula towards the septum resulting in obstruction of the inflow cannula. The cannula was subsequently repositioned. Seven days after VAD implantation, the patient was transferred to the coronary care unit. The remaining post-operative period was uneventful and patient is about to be discharged home.

Peri-operative challenges in the first case included the lack of space between the SRV and the sternum, and the trabeculations in the SRV. The former resulted in mid-basal insertion of the VAD instead of the more apical position that is common for VAD implantation in the left ventricle (Figs. 1 and 2). The latter necessitated resection of multiple trabeculations to prevent obstruction of the inflow cannula. The need for resection of trabeculations was expected in both cases, as pre-operative imaging clearly showed a heavily trabeculated SRV in both patients. This approach has been described previously [5]. In our second case, in addition to resection of trabeculations, a different surgical approach was used because of a relatively dorsolateral position of the SRV. In both patients, the challenging positioning of the inflow cannula could be partially explained by the presence of a tricuspid valve prosthesis, making TEE-guided localisation of optimal inflow cannula position less evident.

In general, SRV patients may have complex cardiac and thoracic anatomy, for example dextrocardia or situs inversus. As these cases demonstrate, anatomical variations in SRV patients require a patient-tailored surgical approach for VAD implantation (median (re)sternotomy versus lateral thoracotomy and upper hemisternotomy). An alternative device position should be considered when lack of space prevents apical implantation of the VAD, and inflow cannula orientation is of paramount importance for unobstructed VAD inflow. Pre-, intra- and post-operative imaging (for example with computed tomography angiography, epicardial/transoesophageal echocardiography, and transthoracic echocardiography, respectively) is crucial to plan and evaluate the operative approach [6].

## Patients with SRV failure are potentially good VAD candidates

Donor hearts are scarce in the Netherlands, a problem which is likely to persist. The three cardiac transplantation centres in the Netherlands together currently perform over 30 transplantations per year [7–9] but the demand is much higher. Furthermore, SRV patients are often unsuitable candidates for cardiac transplantation because of 1) unfavourable anatomy; 2) prior surgical procedures and/or 3) physiology [10]. In the usual LVAD population, right ventricular function is an important clinical predictor for morbidity and mortality after LVAD implantation [11, 12]. However, patients with SRV usually have a preserved function of the subpulmonary left ventricle, which is capable of supporting higher pressures without problems, and may be retrained, even in adult patients [13]. Therefore, selected patients with end-stage SRV failure (see Tab. 1) may be suitable candidates for VAD implantation as destination therapy. This report demonstrates that this is feasible and leads to significant clinical improvement. Data from the INTERMACS registry concerning all reported VAD implantations in patients with CHD, including patients with SRV, show comparable survival rates between CHD and non-CHD patients at 2 years after implantation [14]. However, VAD implantation is also associated with significant complications and requires dedicated teams to optimise results as demonstrated in the current cases. Still, these complications are similar to the complications reported in the LVAD population with normal anatomy [15, 16].

**Table 1:** Medical eligibility criteria and contraindications for VAD implantation as destination therapy in patients with SRV, according to our dedicated team

Major criteria for VAD eligibility (all should apply)	VAD contraindicated if one/more of the following
– End-stage SRV failure (NYHA IIIb–IV, INTERMACS II–IV)	– INTERMACS I
a. Despite optimal medical therapy	– Severe non-cardiac comorbidity with life expectancy <1 year
b. Despite optimal treatment of tricuspid valve regurgitation	– Poor subpulmonary LV function
c. Despite CRT if indicated	– Non-reversible severe kidney dysfunction (eGFR <30 ml/min/1.73m^2^)
d. Despite effort to sustain sinus rhythm	– Active systemic infection
– Ineligible for cardiac transplantation	– Unacceptably high operative risk

*VAD* ventricular assist device, *SRV* systemic right ventricle,* NYHA* New York Heart Association, *INTERMACS* Interagency Registry for Mechanically Assisted Circulatory Support LV left ventricle, *CRT* cardiac resynchronisation therapy, *eGFR* estimated glomerular filtration rate

## Conclusion

In conclusion, VAD implantation as destination therapy should be considered in patients with severe SRV failure. Despite the risk of complications, VAD therapy is a reasonable option in patients with failing SRV but requires a dedicated and experienced team.
